# The Double-Deficient Older Inpatient: Vitamin D and Vitamin B12 Co-Deficiency Identifies the Greatest Cognitive and Affective Burden

**DOI:** 10.3390/nu18183078

**Published:** 2026-09-20

**Authors:** Lucretia Avram, Valer Donca, Tudor Cosma, Diana Moldovan, Corina Radu, Dana Crisan

**Affiliations:** 1 Geriatrics—Gerontology, Department 5—Medical Specicalties, Faculty of Medicine, “Iuliu Hatieganu” University of Medicine and Pharmacy, 400012 Cluj-Napoca, Romaniavalerdonca@gmail.com (V.D.); 2 Regional Institute of Gastroenterology and Hepatology “Prof. Dr. Octavian Fodor”, Faculty of Medicine, “Iuliu Hatieganu” University of Medicine and Pharmacy, 400012 Cluj-Napoca, Romania; 3 Department of Nephrology, Faculty of Medicine, “Iuliu Hatieganu” University of Medicine and Pharmacy, 400012 Cluj-Napoca, Romania; 4 Internal Medicine, Department 4–5th Medical Clinic, Faculty of Medicine, “Iuliu Hatieganu” University of Medicine and Pharmacy, 400012 Cluj-Napoca, Romania

**Keywords:** vitamin D deficiency, vitamin B12 deficiency, co-deficiency, cognitive impairment, frailty, elderly

## Abstract

Background/Objectives: Vitamin D and vitamin B12 deficiencies are both widespread among older adults, yet their combined status is rarely examined within the same cohort and whether the two exposures interact or simply accumulate remains unresolved. This study examined the association between combined vitamin D and B12 status and cognitive, affective, and frailty outcomes in hospitalised older adults, and formally tested for an interaction between the two deficiencies. Methods: This cross-sectional study included 1359 consecutive patients aged 65 years or older admitted for comprehensive geriatric assessment in Cluj-Napoca, Romania, between January 2023 and April 2026, with concurrent serum 25-hydroxyvitamin D and vitamin B12 measurements. Patients were classified as having no deficiency, isolated vitamin D deficiency (<20 ng/mL), isolated vitamin B12 deficiency (<200 pg/mL), or co-deficiency, and outcomes were compared by multivariable regression with a formal interaction term. Results: Co-deficiency affected 96 patients (7.1%). Isolated vitamin D deficiency was independently associated with poorer cognition and greater frailty, while isolated B12 deficiency showed no association with any outcome. Co-deficient patients carried the heaviest cognitive burden of all groups and showed a signal of elevated depressive symptoms that was significant at the primary B12 threshold but attenuated at an alternative threshold, indicating this finding should be interpreted cautiously; no interaction term reached significance. Conclusions: Vitamin D deficiency, alone or combined with B12 deficiency, consistently tracked with poorer cognition and frailty, and co-deficiency marked the subgroup at greatest cognitive and affective risk through an additive rather than synergistic mechanism. These findings support routine vitamin D assessment in geriatric practice, with concurrent B12 deficiency flagging patients who warrant closer attention.

## 1. Introduction

Population ageing has steadily increased the number of older adults living with cognitive impairment, depressive symptomatology, and functional dependency, conditions that substantially reduce quality of life and place growing demands on health and social care systems [1]. Micronutrient status is increasingly recognised as one of the more accessible modifiable contributors to this burden, as deficiencies are common, inexpensive to detect, and potentially correctable [2,3]. Among the micronutrients most frequently implicated in geriatric decline, vitamin D and vitamin B12 have each been studied extensively in isolation, yet their combined status has received comparatively limited systematic attention [3,4].

Vitamin D is obtained through cutaneous synthesis and dietary intake; following hepatic and renal hydroxylation, it acts through the vitamin D receptor, which is widely expressed in the central nervous system and skeletal muscle [5,6]. Age-related declines in cutaneous synthesis and sun exposure leave hospitalised and institutionalised older adults particularly susceptible to deficiency, with reported prevalence frequently exceeding eighty to ninety percent [7,8]. Low serum 25-hydroxyvitamin D concentrations have been associated with poorer cognitive performance, greater physical frailty, and higher mortality [7,9,10]. The most recent Endocrine Society clinical practice guideline continues to support empiric vitamin D supplementation in the oldest old, while recommending against routine biochemical screening [11].

Vitamin B12 acts as an obligatory cofactor for homocysteine remethylation and for the maintenance of myelin integrity [12,13]. Deficiency prevalence rises with age, reflecting atrophic gastritis and the increasing use of medications such as metformin and proton pump inhibitors [14,15]. Low B12 status, most often assessed through total serum concentration, has been associated cross-sectionally with musculoskeletal impairment, depressive symptoms, and cognitive decline [12,16,17]. Evidence from interventional studies, however, remains considerably less consistent than that for vitamin D, and functional biomarkers such as methylmalonic acid are considered more reliable indicators of true cobalamin status than total serum B12 [12,16,18].

Although both micronutrients are frequently deficient in older adults, few studies have examined their combined status within the same cohort, and the nature of their relationship, additive or synergistic, remains insufficiently clarified [19,20]. Co-occurrence of the two deficiencies has been documented across several populations, being associated with shared dietary determinants and with a greater predicted burden of cognitive risk [20,21]. The conceptual framework of cumulative micronutrient insufficiency, rather than isolated deficiency, has gained traction in the recent frailty literature [3,4]. Whether this cumulative risk reflects a genuine biological interaction between vitamin D and vitamin B12, or the coexistence of two independent risk factors acting through mechanistically distinct pathways, remains to be clarified [19,22,23].

The present study examined the combined status of vitamin D and vitamin B12 in a cohort of hospitalised older adults, using a four-group mutually exclusive classification: absence of both deficiencies, isolated vitamin D deficiency, isolated vitamin B12 deficiency, and co-deficiency of both micronutrients. The association of these groups with a broad panel of geriatric outcomes, including cognitive performance, depressive symptoms, physical frailty, and functional dependency, was examined, and a formal interaction term between the two deficiencies was tested separately to distinguish an additive pattern of risk from a synergistic one.

## 2. Materials and Methods

### 2.1. Study Design and Ethical Approval

This cross-sectional observational study was conducted at the Geriatrics Department of the Municipal Clinical Hospital, Cluj-Napoca, Romania. Consecutive patients aged 65 years or older, admitted for a comprehensive geriatric assessment, were enrolled between January 2023 and April 2026. All assessments were completed within the same hospital admission, typically within 48 to 72 h of entry, following a standardised sequence: collection of demographic and clinical history, venous blood sampling, functional evaluation, cognitive screening, and affective assessment. The study was conducted in accordance with the Declaration of Helsinki and was approved by the Institutional Review Board of the Municipal Clinical Hospital Cluj-Napoca (Protocol No. 29/2020, approved on 16 October 2020). Written informed consent was obtained from all participants prior to enrolment.

### 2.2. Participants

Patients were eligible if they were able to complete the full geriatric assessment protocol and had concurrent measurements of serum 25-hydroxyvitamin D and serum vitamin B12. Exclusion criteria comprised acute severe illness, delirium, terminal illness, severe dementia, active psychosis, and major uncorrected sensory impairment likely to compromise reliable completion of the assessment instruments. Severe dementia was identified through a combination of a pre-existing clinical diagnosis documented in the medical record, inability to cooperate with the assessment protocol, and a Montreal Cognitive Assessment score below 10. A total of 1359 patients met the inclusion criteria and were classified into four mutually exclusive groups according to their combined micronutrient status: no deficiency (*n* = 648), isolated vitamin D deficiency (*n* = 528), isolated vitamin B12 deficiency (*n* = 87), and combined vitamin D and B12 deficiency, hereafter referred to as co-deficiency (*n* = 96). Patients were identified in the database by a unique personal code rather than by name.

### 2.3. Biochemical Assessment of Vitamin D and Vitamin B12

All participants underwent a standardised haematological and biochemical panel appropriate for the geriatric population, including a complete blood count, an anaemia panel (serum iron, ferritin, transferrin saturation), C-reactive protein, serum albumin, serum creatinine, and the estimated glomerular filtration rate. Serum 25-hydroxyvitamin D and serum vitamin B12 were measured by electrochemiluminescence immunoassay (ECLIA; Roche Diagnostics, Basel, Switzerland) on a fully automated analyser at the hospital’s central laboratory, using samples collected during the same admission as the clinical assessment. Vitamin D deficiency was defined as a serum concentration below 20 ng/mL, consistent with current Endocrine Society guidance [11] and widely applied in the geriatric literature [7,8]; concentrations of 20–30 ng/mL and above 30 ng/mL were considered insufficient and sufficient, respectively. Vitamin B12 deficiency was defined as a serum concentration below 200 pg/mL, consistent with prior studies in comparable populations [19,24] and with current clinical guidance on the diagnosis of B12 deficiency [14]. Because the international 200 pg/mL threshold has been suggested to under-detect deficiency in some populations [20], a sensitivity analysis using an alternative threshold of 300 pg/mL was additionally performed, with results reported in the Supplementary Material.

### 2.4. Geriatric Assessment

Global cognitive function was assessed with the Montreal Cognitive Assessment (MoCA), a 30-point instrument validated for the detection of mild cognitive impairment in older adults, with lower scores indicating poorer performance [25]. Depressive symptomatology was assessed with the Geriatric Depression Scale (GDS), a self-report instrument developed and validated specifically for older populations [26]. Physical frailty was assessed using two complementary instruments: the Fried frailty phenotype, which classifies individuals according to five physical criteria, namely unintentional weight loss, weakness, exhaustion, slow gait speed, and low physical activity [27], and the Clinical Frailty Scale (CFS), a nine-level, judgement-based global scale [28]. The Edmonton Frail Scale, a multidomain instrument incorporating cognitive, functional, and medical components, was used as an additional frailty measure [29]. Functional status was assessed with a functional dependency scale used in the standardised geriatric assessment of the department, comprising two components. The Activities of Daily Living (ADL) component covered ten basic activity domains (bed mobility, bed-to-chair transfer, mobility within the ward, mobility outside the ward, climbing stairs, dressing, feeding, toilet use, personal hygiene, and bathing or showering), each rated on an ordinal scale from 0 (complete independence) to 8 (total dependence, requiring two assistants or the activity not being feasible), giving a theoretical total score of 0 to 80. The Instrumental Activities of Daily Living (IADL) component covered the eight instrumental domains originally described by Lawton and Brody [30] (telephone use, shopping, food preparation, housekeeping, laundry, transportation, medication management and handling of finances), each rated on a graded ordinal dependency scale ranging from 0 to 2, 3 or 4 according to the domain, giving a theoretical total score of 0 to 23. For both components, higher scores indicate a greater degree of functional dependency. Nutritional status was assessed with the Mini Nutritional Assessment Short Form (MNA-SF) [31] and the Controlling Nutritional Status (CONUT) score [32]. Renal function was estimated using the Chronic Kidney Disease Epidemiology Collaboration (CKD-EPI) equation [33]. All instruments were administered by trained assessors, identically across participants.

### 2.5. Statistical Analysis

Analyses were performed using IBM SPSS Statistics, version 31 (IBM Corp., Armonk, NY, USA). Continuous variables departed from normality on Shapiro–Wilk testing and are reported as median [interquartile range] and compared across the four micronutrient groups using the Kruskal–Wallis test; the associated test statistic (H) was used to calculate epsilon-squared, defined as (H − k + 1)/(N − k), as the accompanying effect size and interpreted against conventional thresholds of 0.01, 0.06 and 0.14 for small, medium and large effects; categorical variables are reported as counts with percentages and compared using the Pearson chi-square test.

The association between micronutrient status and each continuous outcome was examined by multivariable linear regression, with the four-group classification entered as indicator variables and the absence of both deficiencies as the reference category. Model 1 was adjusted for age, body mass index, serum albumin and estimated glomerular filtration rate, all specified a priori; Model 2 additionally for haemoglobin and sex; and Model 3, reported in the Supplementary Material, further for C-reactive protein, which was incompletely recorded. Dichotomous outcomes were analysed by binary logistic regression using the same covariate sets, with mild cognitive impairment defined as a MoCA score below 26, frailty as a Fried score of 3 or more, and severe frailty as a Clinical Frailty Scale score of 7 or more. To distinguish an additive from a synergistic pattern of risk, a separate specification entered the two deficiencies as individual binary terms together with their multiplicative product, adjusted for the Model 1 covariates.

Robustness was assessed in two ways: all analyses were repeated using an alternative vitamin B12 threshold of 300 pg/mL, and the principal models were refitted after rank transformation of the dependent variable. Missing data were handled by complete-case analysis, and the number of valid observations is reported for each instrument and model. All tests were two-sided, with significance set at *p* < 0.05. No adjustment for multiple comparisons was applied; *p* values are therefore unadjusted.

## 3. Results

### 3.1. Study Population and Micronutrient Status

**Figure 1:**
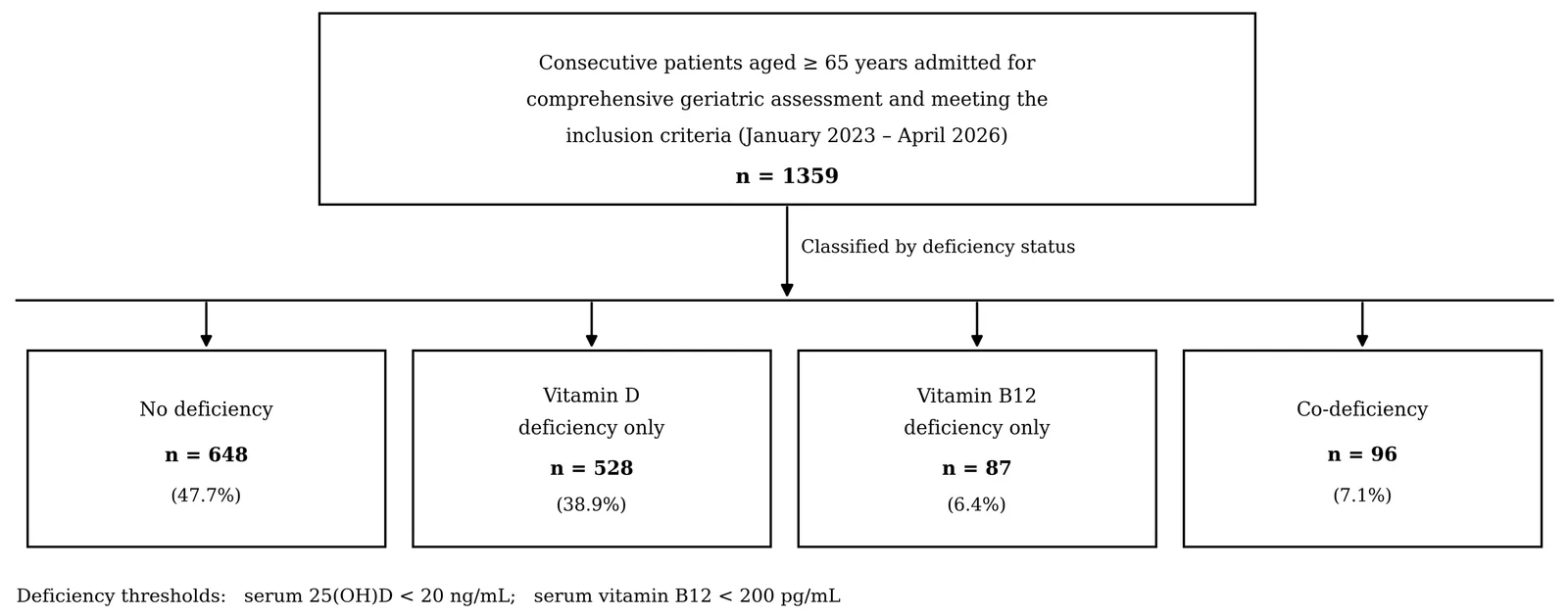
Classification of the study population according to combined vitamin D and vitamin B12 status. Eligibility and exclusion criteria are described in Section 2. Patients were identified by their personal identification number rather than by name, because 16 names were shared by two or more different individuals in the source database. The four groups are mutually exclusive and percentages refer to the 1359 patients with concurrent measurements of both micronutrients.

Of the 1359 patients with concurrent measurements of both micronutrients, 648 (47.7%) had neither deficiency, 528 (38.9%) had isolated vitamin D deficiency, 87 (6.4%) had isolated vitamin B12 deficiency and 96 (7.1%) had both, hereafter co-deficiency (Figure 1). Vitamin D deficiency was therefore present in 624 patients (45.9%) and vitamin B12 deficiency in 183 (13.5%). Median serum 25-hydroxyvitamin D was 31.51 ng/mL [25.42–39.88] in patients without either deficiency and 10.69 ng/mL [7.45–14.50] in those with co-deficiency, while median vitamin B12 fell from 370.49 pg/mL [290.70–523.82] to 156.41 pg/mL [148.00–179.85] across the same two groups (Table 1).

The four groups differed in age, which was approximately three years higher in both vitamin D-deficient groups than in patients without deficiency (81.57 and 81.56 years versus 78.62 years; *p* < 0.001), and in sex distribution, the proportion of women falling from 74.2% in the group without deficiency to 62.5% in the co-deficiency group (χ^2^ = 9.99, degrees of freedom [*df*] = 3, *p* = 0.019). Body mass index did not differ across groups (*p* = 0.524). Serum albumin was lower in the two vitamin D-deficient groups (4.10 g/dL versus 4.30 g/dL; *p* < 0.001) and C-reactive protein correspondingly higher (*p* < 0.001). Haemoglobin was likewise lower in these groups (12.65 and 12.90 g/dL versus 13.20 g/dL; *p* < 0.001). Estimated glomerular filtration rate also differed across groups (*p* = 0.004), although without a consistent gradient: it was lowest in isolated vitamin D deficiency (65.88 mL/min/1.73 m^2^) and highest in co-deficiency (77.64 mL/min/1.73 m). Nutritional status followed the same gradient, with lower MNA-SF and higher CONUT scores in the presence of vitamin D deficiency (both *p* < 0.001).

### 3.2. Geriatric Assessment Across Micronutrient Groups

All seven geriatric instruments differed significantly across the four groups (Table 2). The largest effect sizes were observed for Instrumental Activities of Daily Living (H = 64.97, ε^2^ = 0.089), the Clinical Frailty Scale (H = 103.77, ε^2^ = 0.075) and basic Activities of Daily Living (H = 93.02, ε^2^ = 0.067), all of medium magnitude. Cognitive performance, physical frailty and the Edmonton Frail Scale showed comparable and smaller effects (ε^2^ = 0.044, 0.043 and 0.043, respectively). Depressive symptomatology was the weakest signal in this analysis, reaching significance only marginally and with a negligible effect size (H = 10.51, *p* = 0.015, ε^2^ = 0.006).

The distribution across groups was consistent in direction. Patients with co-deficiency had the lowest median MoCA score of the four groups (18.0 [13.5–23.0] versus 23.0 [18.0–27.0] in patients without deficiency) and the highest median Geriatric Depression Scale score (14.0 [9.0–19.0] versus 11.0 [6.0–17.0]). Patients with isolated vitamin B12 deficiency were, on every instrument, closer to the group without deficiency than to either vitamin D-deficient group.

### 3.3. Multivariable Linear Regression

**Figure 2:**
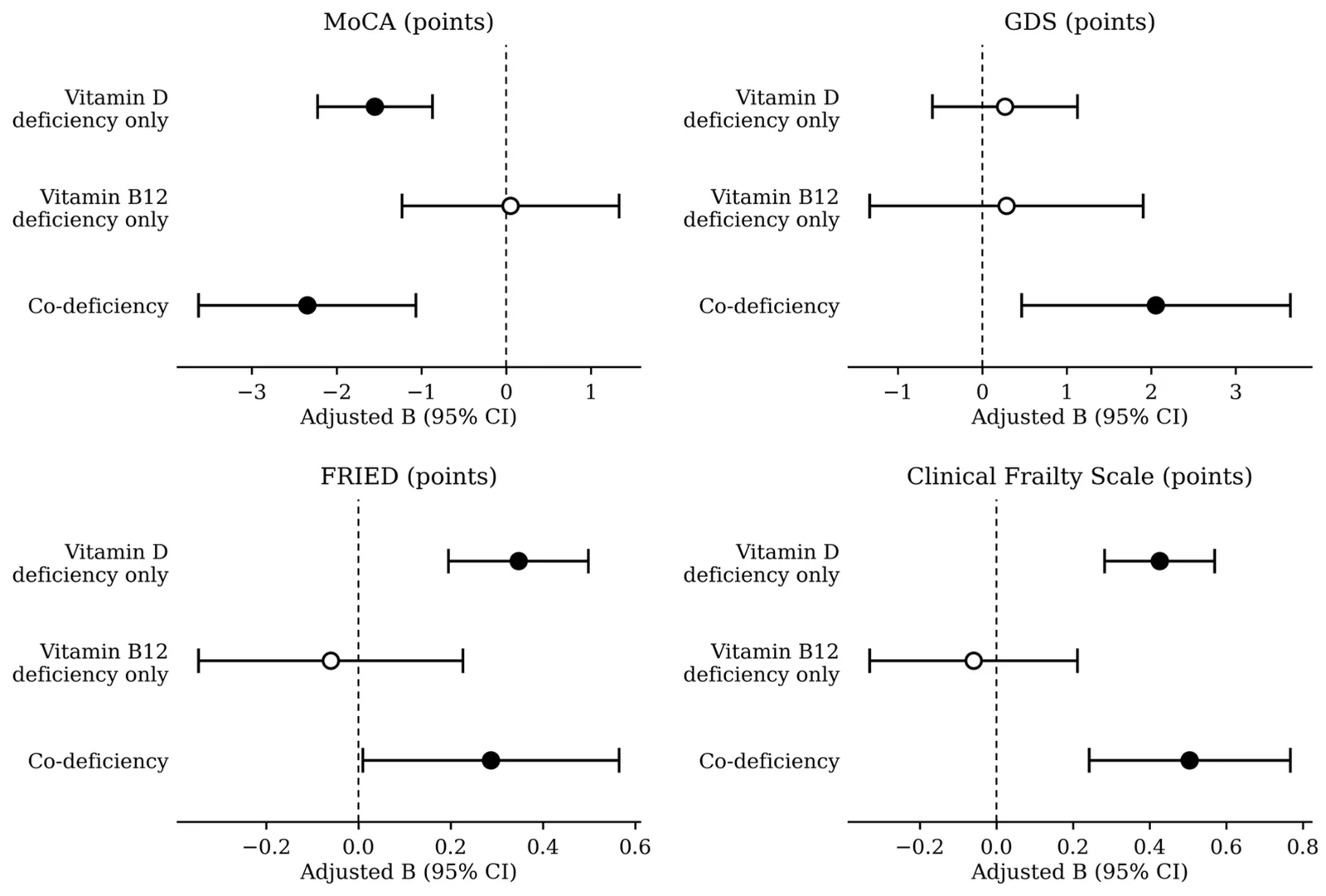
Adjusted associations between micronutrient deficiency status and geriatric outcomes. Points represent unstandardized regression coefficients (B) with 95% confidence intervals, derived from multivariable linear regression adjusted for age, body mass index, serum albumin and estimated glomerular filtration rate (Model 1). The reference category is the absence of both deficiencies. Filled circles denote associations that reached statistical significance (*p* < 0.05); open circles denote non-significant associations. Horizontal bars represent 95% confidence intervals, and the vertical dashed line marks the null value of zero. Higher MoCA scores denote better cognitive performance, whereas higher GDS, FRIED and Clinical Frailty Scale scores denote greater impairment. Note the pattern for GDS, in which neither deficiency alone is associated with the outcome while their combination is.

After adjustment for age, body mass index, serum albumin and estimated glomerular filtration rate, isolated vitamin D deficiency was associated with a MoCA score 1.549 points lower than in patients without deficiency (95% confidence interval [CI] −2.225 to −0.873; *p* < 0.001), and co-deficiency with a reduction of 2.348 points (95% CI −3.630 to −1.067; *p* < 0.001). Isolated vitamin B12 deficiency showed no association (unstandardized regression coefficient [B] = 0.050; *p* = 0.939). The same pattern held for physical frailty, where isolated vitamin D deficiency was associated with a Fried score 0.347 points higher (95% CI 0.195 to 0.498; *p* < 0.001) and co-deficiency with 0.287 points (95% CI 0.009 to 0.565; *p* = 0.043), and for the Clinical Frailty Scale, with increments of 0.426 (95% CI 0.282 to 0.569) and 0.504 points (95% CI 0.242 to 0.767), respectively, both *p* < 0.001 (Table 3, Figure 2).

Depressive symptomatology followed a different pattern. Neither deficiency alone was associated with the Geriatric Depression Scale score, whereas their combination was: co-deficiency corresponded to a score 2.055 points higher than the reference group (95% CI 0.464 to 3.647; *p* = 0.011), an estimate that strengthened after further adjustment for haemoglobin and sex (B = 2.339; 95% CI 0.757 to 3.921; *p* = 0.004). This association proved sensitive to the choice of B12 threshold, however, and did not persist when the cut-off was set at 300 pg/mL. Isolated vitamin B12 deficiency was not associated with any of the four outcomes in either model.

Additional adjustment for haemoglobin and sex left all estimates essentially unchanged (Table 3), and the explained variance was largely unaffected (R^2^ = 0.288 and 0.290 for MoCA, 0.256 and 0.264 for the Fried score, and 0.354 and 0.360 for the Clinical Frailty Scale, in Models 1 and 2 respectively). The corresponding figures for the Geriatric Depression Scale were substantially lower (R^2^ = 0.050 and 0.078), indicating that the covariates explain only a small fraction of the variance in depressive symptomatology. In the most extensively adjusted specification, which additionally included C-reactive protein and was based on a reduced sample of 970 patients, the association between co-deficiency and MoCA was attenuated and fell marginally short of significance (B = −1.517; 95% CI −3.077 to 0.043; *p* = 0.057), while all other associations were retained (Supplementary Table S1).

### 3.4. Binary Logistic Regression

When the outcomes were dichotomised, the associations with frailty persisted while those with cognition did not (Table 4). Isolated vitamin D deficiency was associated with frailty defined as a Fried score of three or more (odds ratio [OR] 1.785; 95% CI 1.367 to 2.331; *p* < 0.001) and with severe frailty defined as a Clinical Frailty Scale score of seven or more (OR 2.150; 95% CI 1.600 to 2.888; *p* < 0.001). Co-deficiency carried comparable odds of frailty (OR 1.714; 95% CI 1.039 to 2.826; *p* = 0.035) and the highest odds of severe frailty of any group (OR 2.281; 95% CI 1.356 to 3.838; *p* = 0.002), the latter estimate increasing to 2.335 (95% CI 1.386 to 3.936; *p* = 0.001) after adjustment for haemoglobin and sex. For mild cognitive impairment, defined as a MoCA score below 26, no group reached significance, the estimate for co-deficiency being 1.670 (95% CI 0.852 to 3.274; *p* = 0.135). This outcome was present in 73.0% of the analysed sample, so that dichotomisation discarded much of the variation captured by the continuous MoCA score. Isolated vitamin B12 deficiency was not associated with any of the three dichotomous outcomes.

### 3.5. Interaction Between the Two Deficiencies

In the specification in which the two deficiencies entered as separate binary terms together with their product, the interaction term did not reach significance for any of the four principal outcomes, either at the primary vitamin B12 threshold of 200 pg/mL (*p* = 0.360 for MoCA, 0.196 for the Geriatric Depression Scale, 0.998 for the Fried score and 0.472 for the Clinical Frailty Scale) or at the alternative threshold of 300 pg/mL (*p* = 0.303, 0.062, 0.362 and 0.526, respectively). The closest approach to significance was the term for depressive symptomatology at the higher threshold, which nonetheless remained above the conventional level (Supplementary Table S2). With 96 patients in the co-deficiency cell, however, the power to detect a departure from additivity was limited, and these results should be read as an absence of evidence for a multiplicative effect rather than as evidence of its absence.

### 3.6. Sensitivity Analyses

Repeating the analyses with vitamin B12 deficiency defined as a concentration below 300 pg/mL redistributed the cohort considerably, the co-deficiency group increasing from 96 to 278 patients. The associations with cognition, physical frailty and the Clinical Frailty Scale were confirmed at this threshold and were of similar magnitude, whereas the association between co-deficiency and depressive symptomatology was attenuated and no longer significant (B = 0.617; 95% CI −0.485 to 1.719; *p* = 0.272). The group comparisons on the geriatric instruments were likewise unchanged, with effect sizes differing by no more than 0.004 between the two thresholds (Supplementary Table S3).

Refitting the principal regression models after rank transformation of the dependent variable confirmed the direction and the significance status of eleven of the twelve exposure coefficients. The single exception was the coefficient for co-deficiency in the Fried model, which moved marginally above the conventional threshold (*p* = 0.043 in the untransformed model and *p* = 0.052 after rank transformation), indicating that this particular association is the most fragile of those reported (Supplementary Table S4).

## 4. Discussion

In this cross-sectional study of hospitalised older adults, vitamin D status emerged as the dominant micronutrient signal. Isolated vitamin D deficiency was associated with poorer performance on the continuous MoCA score and with more pronounced physical frailty, although this association did not extend to the binary classification of mild cognitive impairment. Isolated vitamin B12 deficiency, in contrast, left no discernible statistical trace across any of the domains assessed. Once the two deficiencies coexisted, however, co-deficient patients showed the most pronounced cognitive decline of all groups and were the only subgroup with significantly elevated depressive symptoms. Formal testing of an interaction term between the two exposures did not support a multiplicative relationship. The data instead describe a gradual accumulation of risk, in which each deficiency appears to contribute separately to the clinical picture, rather than through a mutual amplification of effects. This apparent accumulation of risk should be interpreted with some caution, since vitamin D-deficient subgroups also displayed lower albumin, higher C-reactive protein and less favourable nutritional indices, raising the possibility that part of the observed associations reflects underlying inflammation or malnutrition rather than micronutrient status itself. This framing is consistent with a broader conceptual shift in the frailty literature, which increasingly treats cumulative micronutrient insufficiency, rather than any single deficiency, as the more informative risk marker [3,4].

The independent association between vitamin D deficiency and unfavourable cognitive and frailty outcomes sits comfortably within a substantial body of prior evidence. Serum 25(OH)D concentrations decline in a graded fashion with frailty severity [7] and rising concentrations over time accompany a more favourable frailty trajectory, lending weight to a temporal rather than purely cross-sectional relationship [10]. A comparable dissociation emerges on the cognitive side, with vitamin D, but not B12 correlating inversely with cognitive frailty in large community-dwelling cohorts [34,35] and vitamin D deficiency independently predicting mortality where B12 and folate do not [9]. This is consistent with survey and consensus data identifying vitamin D status as a persistent, high-priority concern in the clinical assessment of ageing adults [36] and together these observations position vitamin D, rather than B12, as the principal prognostic axis among the micronutrients most commonly assessed in older adults [37].

The absence of any association for isolated vitamin B12 deficiency should not be read as evidence that this micronutrient lacks clinical relevance; it more plausibly reflects the limitations of total serum B12 as a proxy for true functional cobalamin status. A non-linear, U-shaped relationship with cognitive impairment has been described for serum B12 [22] and functional markers such as methylmalonic acid and homocysteine have repeatedly outperformed total serum B12 as predictors of cognitive impairment [38,39,40,41,42,43]. Because the present analysis relied exclusively on total serum B12, some degree of misclassification of true cobalamin status cannot be excluded, a point taken up further in the Limitations.

The hypothesis of a mutually reinforcing relationship between the two deficiencies was not borne out by our data. This hypothesis originates from a single study, in patients with autoimmune thyroiditis, where a correlation between vitamin D and B12 concentrations was interpreted as a possible sign of metabolic synergy [19]; being the only source proposing this specific interaction, we considered it worth testing formally despite the different clinical context. A multiplicative interaction term, examined across four principal outcomes and two alternative B12 thresholds, did not reach significance in any model, indicating that such an interaction does not appear to operate through vitamin D in our cohort, without ruling out a genuine biological interaction. This asymmetry mirrors a broader pattern in the supplementation literature, where B-complex interventions have generally shown more consistent cognitive signals than vitamin D alone [44]. This picture is not, however, uniformly favourable: broader systematic reviews of B-vitamin supplementation, including folic acid, have generally found no consistent benefit for cognitive performance or decline in older adults [45,46], suggesting that the folate–B12 relationship observed in observational cohorts may not readily translate into a modifiable therapeutic target. An alternative explanation comes from the Framingham cohort, where the cognitive benefit of favourable B12 status becomes most apparent among individuals with elevated folate status [47], suggesting that the more relevant biological partner for vitamin B12 may be folate rather than vitamin D, a nutrient our database did not capture and one we aim to incorporate in future studies.

The pattern observed for depressive symptomatology, absent for either deficiency alone yet present for their combination, lends itself to a plausible, if speculative, mechanistic explanation. Low vitamin B12 status has been linked to a higher four-year incidence of depression in institutionalised older adults [2]. Vitamin D and the B-vitamins are thought to act through distinct pathways: the former via serotonin and calcium signalling, consistent with the wide central expression of the vitamin D receptor [48], the latter via homocysteine-related neurotoxicity [17]. Two independent mechanisms converging on the same outcome would therefore be expected to produce a stepwise rather than multiplicative increase in risk. This finding was, however, threshold-dependent (see Limitations) and should be regarded as exploratory.

From a clinical standpoint, the near-universal prevalence of vitamin D deficiency reported in other cohorts of hospitalised or institutionalised older adults offers a useful frame for interpreting our own findings. In comparable frail geriatric cohorts, 87–96% of unsupplemented individuals present with inadequate concentrations [8,49], a prevalence high enough that some authors argue for setting aside routine biochemical testing before supplementation [8]. This position is echoed by the most recent Endocrine Society guideline, which recommends against systematic 25(OH)D screening while still supporting empiric supplementation in adults aged 75 years and older [11]. For vitamin B12, current guidance instead favours a more selective, risk-factor-based approach to case-finding [50,51], given the well-documented interference of metformin and proton pump inhibitors with cobalamin absorption [52,53].

Set against this evidence, the overall prevalence of vitamin D deficiency observed in the present cohort was considerably lower: 624 of 1359 patients (45.9%) had serum 25(OH)D concentrations below 20 ng/mL. This is well below near-universal rates reported in frail hospitalised or institutionalised cohorts [8,49,54], though broadly consistent with the wider range reported across less selectively frail populations [2]. Several features of the present cohort plausibly account for this gap. Our inclusion criteria captured the full spectrum of consecutive admissions rather than samples restricted to severely frail or bedridden patients, and even patients free of either deficiency had a median Clinical Frailty Scale score corresponding to mild rather than advanced frailty [8]. The gap also narrows once insufficiency is considered: the lower quartile of serum 25(OH)D among patients classified as deficiency-free still fell within the insufficient range (20–30 ng/mL), under the broader definitions used in several comparator studies [36,37]. Finally, recruitment spanning nearly three years likely averaged out winter-driven peaks in deficiency prevalence seen in single-season surveys. Together, these considerations suggest the discrepancy with near-universal figures elsewhere reflects case-mix, threshold definition and sampling season, rather than a genuinely healthier population.

Taken together, these findings support the view that vitamin D deficiency deserves clinical priority in hospitalised older adults, irrespective of B12 status. The simultaneous presence of both deficiencies nonetheless remains a useful signal, identifying the subgroup at greatest risk of cognitive and affective decline, even though the underlying mechanism appears additive rather than synergistic. Future work explicitly modelling folate alongside these two micronutrients [55], and incorporating musculoskeletal outcomes linked elsewhere to B12-specific functional biomarkers [16], would help clarify whether jointly correcting both deficiencies yields measurable clinical benefit.

Several limitations should be considered when interpreting these findings. The cross-sectional design precludes causal inference. Although longitudinal evidence elsewhere suggests that vitamin D trajectories parallel frailty transitions over time [10], no equivalent within-person data were available in this cohort. Vitamin B12 status was captured through total serum concentration rather than through functional biomarkers such as methylmalonic acid or holotranscobalamin, both considered more sensitive indicators of true cobalamin status [12,16,18]; this may partly explain the null findings observed for isolated B12 deficiency. Serum folate was not measured, which precluded formal evaluation of the folate-dependent interaction reported elsewhere for vitamin B12 and cognitive decline [47]. The association between co-deficiency and depressive symptomatology was attenuated at the alternative 300 pg/mL B12 threshold, suggesting this particular finding warrants some caution pending replication. The number of valid observations varied across instruments and was comparatively limited for the Instrumental Activities of Daily Living scale, which may have reduced statistical power for this outcome. Inflammation and nutritional status are also plausible confounders of the associations reported here: C-reactive protein could only be included in Model 3 for a reduced subset of 970 patients owing to incomplete recording, so residual confounding by inflammation or malnutrition, only partially addressed by this adjustment, cannot be excluded. The exclusion of patients with severe dementia, identified through a combination of prior clinical diagnosis, inability to cooperate with the assessment protocol, and a MoCA score below 10, likely narrowed the cognitive range of the sample toward its higher end. Participants with the most advanced cognitive impairment, in whom the effects of micronutrient deficiency might be expected to be most pronounced, were therefore systematically underrepresented, a form of selection bias that would tend to attenuate rather than inflate the associations reported here. The present findings may consequently understate the true burden of vitamin D and B12 deficiency among older adults with more severe cognitive impairment, to whom the results cannot be directly generalised. Finally, the study was conducted at a single geriatric centre, and the findings may not extend directly to non-hospitalised or community-dwelling older populations.

## 5. Conclusions

In this cohort of hospitalised older adults, vitamin D deficiency, whether isolated or combined with vitamin B12 deficiency, was associated with poorer cognitive performance on continuous measures and with greater physical frailty, while isolated vitamin B12 deficiency showed no independent association with any outcome examined. Co-deficiency of the two micronutrients was linked to the most pronounced cognitive impairment observed across all groups and showed a signal of elevated depressive symptomatology that was not consistent across the B12 thresholds tested and should therefore be regarded as preliminary; the relationship between the two deficiencies overall appeared cumulative rather than mutually reinforcing. These findings suggest that vitamin D status should remain a clinical priority in geriatric assessment, while the presence of concurrent B12 deficiency may help identify patients at particularly elevated risk of cognitive and affective decline. Prospective studies incorporating functional B12 biomarkers and folate status would help clarify whether targeted correction of combined deficiency translates into measurable clinical benefit. From a healthcare policy perspective, these findings support embedding vitamin D assessment within routine comprehensive geriatric assessment protocols for hospitalised older adults, given its consistent association with cognitive and frailty outcomes in this large, consecutively recruited cohort. Rather than advocating universal screening for both micronutrients, a more resource-efficient approach may be to prioritise systematic vitamin D testing while reserving B12 assessment for patients with recognised risk factors such as metformin or proton-pump-inhibitor use, consistent with current clinical guidance. Identifying co-deficient patients at admission could nonetheless help geriatric services flag a subgroup at elevated risk of cognitive and affective decline for closer monitoring, offering a low-cost extension of existing assessment pathways rather than a new screening programme.

## Figures and Tables

**Table 1:** Baseline characteristics of the study population according to vitamin D and vitamin B12 status.

Parameter	No Deficiency (*n* = 648)	VitD Deficiency Only (*n* = 528)	B12 Deficiency Only (*n* = 87)	Co-Deficiency (*n* = 96)	*p*
Vitamin D (ng/mL)	31.51 [25.42–39.88]	12.04 [8.87–15.72]	29.65 [25.23–35.10]	10.69 [7.45–14.50]	<0.001
Vitamin B12 (pg/mL)	370.49 [290.70–523.82]	352.51 [278.16–485.99]	152.10 [148.00–179.52]	156.41 [148.00–179.85]	<0.001
Age (years)	78.62 [73.07–84.61]	81.57 [75.98–86.18]	78.96 [74.95–85.18]	81.56 [76.46–86.23]	<0.001
Female sex, *n* (%)	481 (74.2)	365 (69.1)	55 (63.2)	60 (62.5)	0.019
Body mass index (kg/m^2^)	28.41 [24.44–32.38]	27.80 [23.84–32.45]	28.51 [25.82–31.93]	29.20 [24.74–33.49]	0.524
Serum albumin (g/dL)	4.30 [4.00–4.60]	4.10 [3.70–4.40]	4.35 [4.00–4.60]	4.10 [3.70–4.40]	<0.001
eGFR (mL/min/1.73 m^2^)	70.89 [52.51–86.63]	65.88 [44.85–86.09]	74.42 [54.97–86.44]	77.64 [52.91–93.11]	0.004
Haemoglobin (g/dL)	13.20 [12.03–14.20]	12.65 [11.20–14.00]	13.10 [12.00–14.00]	12.90 [11.20–14.00]	<0.001
C-reactive protein (mg/dL)	0.34 [0.14–0.96]	0.50 [0.19–2.12]	0.37 [0.15–1.12]	0.57 [0.17–1.59]	<0.001
MNA-SF (0–14)	11.00 [9.00–13.00]	10.00 [8.00–12.00]	11.00 [9.00–13.00]	11.00 [9.00–13.00]	<0.001
CONUT (0–12)	1.00 [0.00–2.00]	2.00 [1.00–3.00]	1.00 [0.00–2.75]	2.00 [1.00–3.00]	<0.001

Continuous variables are presented as median [interquartile range] and compared using the Kruskal–Wallis test (*df* = 3); sex is presented as *n* (%) and compared using the Pearson chi-square test (χ^2^ = 9.99, *df* = 3). Vitamin D deficiency: <20 ng/mL; vitamin B12 deficiency: <200 pg/mL. Abbreviations: eGFR—estimated glomerular filtration rate; MNA-SF—Mini Nutritional Assessment Short Form; CONUT—Controlling Nutritional Status score.

**Table 2:** Geriatric assessment scores according to vitamin D and vitamin B12 status.

Instrument	No Deficiency	VitD Deficiency Only	B12 Deficiency Only	Co-Deficiency	H	*p*	ε^2^
MoCA (0–30)	23.0 [18.0–27.0]	20.0 [14.0–25.0]	22.0 [16.0–26.0]	18.0 [13.5–23.0]	59.18	<0.001	0.044
GDS (0–30)	11.0 [6.0–17.0]	13.0 [7.0–18.0]	11.0 [6.0–18.5]	14.0 [9.0–19.0]	10.51	0.015	0.006
FRIED (0–5)	2.0 [1.0–3.0]	3.0 [2.0–4.0]	2.0 [1.0–3.0]	3.0 [2.0–4.0]	61.11	<0.001	0.043
Clinical Frailty Scale (1–9)	5.0 [4.0–6.0]	6.0 [5.0–7.0]	6.0 [4.0–7.0]	6.0 [5.3–7.0]	103.77	<0.001	0.075
Edmonton Frail Scale (0–17)	8.0 [5.0–10.0]	9.0 [7.0–11.0]	8.0 [6.0–9.0]	9.0 [7.0–10.0]	57.41	<0.001	0.043
ADL (0–80)	1.0 [0.0–8.0]	6.0 [1.0–20.3]	1.0 [0.0–9.8]	6.0 [0.0–26.0]	93.02	<0.001	0.067
IADL (0–23)	7.0 [1.0–15.0]	15.0 [7.0–21.0]	5.0 [1.0–14.5]	12.0 [4.3–18.0]	64.97	<0.001	0.089

Values are presented as median [interquartile range] and compared using the Kruskal–Wallis test (*df* = 3); H is the test statistic adjusted for ties and ε^2^ the epsilon-squared effect size, defined in Section 2.5. Valid observations per instrument: MoCA 1279; GDS 1213; FRIED 1351; Clinical Frailty Scale 1355; Edmonton Frail Scale 1283; ADL 1344; IADL 704. Higher MoCA scores indicate better cognitive performance; higher scores on all other instruments indicate greater impairment or dependency. The Kruskal–Wallis test addresses the global hypothesis that at least one group differs; covariate-adjusted contrasts against the reference group are reported in Table 3 and Table 4. Abbreviations: MoCA—Montreal Cognitive Assessment; GDS—Geriatric Depression Scale; FRIED—Fried frailty phenotype; ADL—Activities of Daily Living; IADL—Instrumental Activities of Daily Living.

**Table 3:** Multivariable linear regression analyses of micronutrient status and geriatric outcomes.

Outcome	Exposure Group	Model 1 B (95% CI)	*p*	Model 2 B (95% CI)	*p*
MoCA	VitD deficiency only	−1.549 (−2.225–−0.873)	<0.001	−1.583 (−2.259–−0.907)	<0.001
	B12 deficiency only	0.050 (−1.230–1.331)	0.939	0.043 (−1.238–1.324)	0.947
	Co-deficiency	−2.348 (−3.630–−1.067)	<0.001	−2.399 (−3.682–−1.116)	<0.001
GDS	VitD deficiency only	0.266 (−0.592–1.124)	0.543	0.380 (−0.469–1.229)	0.380
	B12 deficiency only	0.285 (−1.335–1.904)	0.730	0.510 (−1.100–2.120)	0.534
	Co-deficiency	2.055 (0.464–3.647)	0.011	2.339 (0.757–3.921)	0.004
FRIED	VitD deficiency only	0.347 (0.195–0.498)	<0.001	0.353 (0.201–0.504)	<0.001
	B12 deficiency only	−0.060 (−0.347–0.226)	0.679	−0.038 (−0.324–0.248)	0.795
	Co-deficiency	0.287 (0.009–0.565)	0.043	0.311 (0.034–0.589)	0.028
Clinical Frailty Scale	VitD deficiency only	0.426 (0.282–0.569)	<0.001	0.434 (0.291–0.577)	<0.001
	B12 deficiency only	−0.060 (−0.331–0.211)	0.664	−0.036 (−0.306–0.235)	0.796
	Co-deficiency	0.504 (0.242–0.767)	<0.001	0.533 (0.271–0.795)	<0.001

B—unstandardized regression coefficient; CI—confidence interval. Reference category: no deficiency. Model 1 adjusted for age, body mass index, serum albumin and eGFR. Model 2 additionally adjusted for haemoglobin and sex (male sex as the indicator category). Sample sizes and explained variance: MoCA *n* = 1246 (R^2^ = 0.288 and 0.290); GDS *n* = 1185 (R^2^ = 0.050 and 0.078); FRIED *n* = 1292 (R^2^ = 0.256 and 0.264); Clinical Frailty Scale *n* = 1294 (R^2^ = 0.354 and 0.360) for Models 1 and 2 respectively. Model 3, additionally adjusted for C-reactive protein, and the complete coefficients for all covariates are provided in Supplementary Table S1. Abbreviations: eGFR—estimated glomerular filtration rate; MoCA—Montreal Cognitive Assessment; GDS—Geriatric Depression Scale; FRIED—Fried frailty phenotype.

**Table 4:** Binary logistic regression analyses of micronutrient status, cognitive impairment and frailty.

Outcome	Exposure Group	Model 1 OR (95% CI)	*p*	Model 2 OR (95% CI)	*p*
Mild cognitive impairment	VitD deficiency only	1.230 (0.894–1.692)	0.204	1.249 (0.907–1.722)	0.173
MoCA < 26; 73.0%; *n* = 1246	B12 deficiency only	0.658 (0.382–1.132)	0.130	0.668 (0.388–1.151)	0.146
	Co-deficiency	1.670 (0.852–3.274)	0.135	1.723 (0.876–3.391)	0.115
Frailty	VitD deficiency only	1.785 (1.367–2.331)	<0.001	1.801 (1.378–2.354)	<0.001
FRIED ≥ 3; 55.2%; *n* = 1292	B12 deficiency only	1.007 (0.613–1.657)	0.977	1.030 (0.626–1.695)	0.908
	Co-deficiency	1.714 (1.039–2.826)	0.035	1.758 (1.064–2.906)	0.028
Severe frailty	VitD deficiency only	2.150 (1.600–2.888)	<0.001	2.167 (1.612–2.914)	<0.001
CFS ≥ 7; 33.8%; *n* = 1294	B12 deficiency only	1.280 (0.709–2.311)	0.414	1.309 (0.725–2.364)	0.371
	Co-deficiency	2.281 (1.356–3.838)	0.002	2.335 (1.386–3.936)	0.001

Reference category: no deficiency; Model 1 was adjusted for age, body mass index, serum albumin and eGFR and Model 2 additionally for haemoglobin and sex, giving Nagelkerke R^2^ values in Models 1 and 2 of 0.288 and 0.289 for mild cognitive impairment, 0.239 and 0.243 for frailty and 0.323 and 0.325 for severe frailty; pre-frailty or frailty (FRIED ≥ 1) was not modelled because its prevalence of 90.9% renders odds ratio estimation unstable. Abbreviations: OR—odds ratio; CI—confidence interval; eGFR—estimated glomerular filtration rate; MoCA—Montreal Cognitive Assessment; FRIED—Fried frailty phenotype; CFS—Clinical Frailty Scale.

## Data Availability

The dataset used during the current study is available from the corresponding authors upon reasonable request.

## Supplementary Materials

The following supporting information can be downloaded at https://www.mdpi.com/article/10.3390/nu18183078/s1. Table S1. Complete coefficients of the multivariable linear regression models; Table S2. Multiplicative interaction between vitamin D and vitamin B12 deficiency; Table S3. Sensitivity analysis using the alternative vitamin B12 threshold of 300 pg/mL; Table S4. Confirmation of the multivariable models using rank-transformed outcomes.
