# Supplementary material for: The Double-Deficient Older Inpatient: Vitamin D and Vitamin B12 Co-Deficiency Identifies the Greatest Cognitive and Affective Burden
Source: Nutrients. 2026 Sep 20;18(18):3078. doi: 10.3390/nu18183078 (PMC13610524; doi:10.3390/nu18183078)
Supplement: Supplementary file 1 [file nutrients-18-03078-s001.zip › nutrients-4535775-supplementary.pdf]

Supplementary Material

Vitamin D and vitamin B12 co-deficiency in hospitalised older adults

Supplementary Table S1. Complete coefficients of the multivariable linear regression models

MoCA

Parameter	Model 1 B (95% CI)	p	Model 2 B (95% CI)	p	Model 3 B (95% CI)	p
Age (years)	−0.357 (−0.403 – −0.311)	<0.001	−0.359 (−0.406 – −0.313)	<0.001	−0.365 (−0.418 – −0.311)	<0.001
Body mass index (kg/m²)	0.049 (−0.001 – 0.098)	0.053	0.056 (0.007 – 0.106)	0.026	0.045 (−0.011 – 0.100)	0.114
Serum albumin (g/dL)	1.825 (1.161 – 2.489)	<0.001	2.093 (1.359 – 2.827)	<0.001	2.178 (1.286 – 3.069)	<0.001
eGFR (mL/min/1.73 m²)	−0.004 (−0.018 – 0.010)	0.573	−0.002 (−0.017 – 0.012)	0.735	−0.002 (−0.018 – 0.014)	0.785
Haemoglobin (g/dL)	—	—	−0.147 (−0.325 – 0.031)	0.105	−0.163 (−0.362 – 0.035)	0.107
Male sex	—	—	0.386 (−0.303 – 1.076)	0.272	0.231 (−0.561 – 1.023)	0.567
C-reactive protein (mg/dL)	—	—	—	—	−0.020 (−0.123 – 0.083)	0.697
VitD deficiency only	−1.549 (−2.225 – −0.873)	<0.001	−1.583 (−2.259 – −0.907)	<0.001	−1.346 (−2.114 – −0.577)	<0.001
B12 deficiency only	0.050 (−1.230 – 1.331)	0.939	0.043 (−1.238 – 1.324)	0.947	0.145 (−1.390 – 1.680)	0.853
Co-deficiency	−2.348 (−3.630 – −1.067)	<0.001	−2.399 (−3.682 – −1.116)	<0.001	−1.517 (−3.077 – 0.043)	0.057
n; R^2	1,246; 0.288		1,246; 0.290		970; 0.287	

GDS

Parameter	Model 1 B (95% CI)	p	Model 2 B (95% CI)	p	Model 3 B (95% CI)	p
Age (years)	0.124 (0.065 – 0.182)	<0.001	0.123 (0.065 – 0.181)	<0.001	0.125 (0.057 – 0.192)	<0.001
Body mass index (kg/m²)	0.022 (−0.040 – 0.084)	0.479	−0.004 (−0.067 – 0.059)	0.889	0.003 (−0.066 – 0.072)	0.932
Serum albumin (g/dL)	−1.287 (−2.131 – −0.442)	0.003	−1.530 (−2.449 – −0.612)	0.001	−1.712 (−2.836 – −0.589)	0.003
eGFR (mL/min/1.73 m²)	−0.015 (−0.032 – 0.002)	0.092	−0.011 (−0.029 – 0.007)	0.195	−0.004 (−0.025 – 0.016)	0.663
Haemoglobin (g/dL)	—	—	0.068 (−0.156 – 0.291)	0.552	0.035 (−0.216 – 0.286)	0.785
Male sex	—	—	−2.617 (−3.482 – −1.752)	<0.001	−2.537 (−3.539 – −1.534)	<0.001
C-reactive protein (mg/dL)	—	—	—	—	−0.061 (−0.191 – 0.070)	0.362
VitD deficiency only	0.266 (−0.592 – 1.124)	0.543	0.380 (−0.469 – 1.229)	0.380	0.672 (−0.293 – 1.637)	0.172
B12 deficiency only	0.285 (−1.335 – 1.904)	0.730	0.510 (−1.100 – 2.120)	0.534	0.360 (−1.595 – 2.316)	0.718

Parameter	Model 1 B (95% CI)	p	Model 2 B (95% CI)	p	Model 3 B (95% CI)	p
Co-deficiency	2.055 (0.464 – 3.647)	0.011	2.339 (0.757 – 3.921)	0.004	2.525 (0.588 – 4.461)	0.011
n; R^2	1,185; 0.050		1,185; 0.078		914; 0.079	

FRIED

Parameter	Model 1 B (95% CI)	p	Model 2 B (95% CI)	p	Model 3 B (95% CI)	p
Age (years)	0.058 (0.048 – 0.068)	<0.001	0.057 (0.047 – 0.067)	<0.001	0.058 (0.046 – 0.070)	<0.001
Body mass index (kg/m²)	−0.003 (−0.014 – 0.008)	0.652	−0.004 (−0.015 – 0.007)	0.512	−0.004 (−0.016 – 0.009)	0.568
Serum albumin (g/dL)	−0.645 (−0.792 – −0.499)	<0.001	−0.597 (−0.759 – −0.435)	<0.001	−0.579 (−0.773 – −0.385)	<0.001
eGFR (mL/min/1.73 m²)	−0.004 (−0.007 – −0.001)	0.020	−0.003 (−0.006 – 0.000)	0.045	−0.003 (−0.006 – 0.001)	0.154
Haemoglobin (g/dL)	—	—	−0.034 (−0.073 – 0.006)	0.094	−0.025 (−0.069 – 0.019)	0.264
Male sex	—	—	−0.233 (−0.386 – −0.080)	0.003	−0.210 (−0.385 – −0.035)	0.019
C-reactive protein (mg/dL)	—	—	—	—	0.019 (−0.003 – 0.041)	0.094
VitD deficiency only	0.347 (0.195 – 0.498)	<0.001	0.353 (0.201 – 0.504)	<0.001	0.407 (0.236 – 0.578)	<0.001
B12 deficiency only	−0.060 (−0.347 – 0.226)	0.679	−0.038 (−0.324 – 0.248)	0.795	0.000 (−0.341 – 0.341)	0.999
Co-deficiency	0.287 (0.009 – 0.565)	0.043	0.311 (0.034 – 0.589)	0.028	0.343 (0.011 – 0.675)	0.043
n; R^2	1,292; 0.256		1,292; 0.264		1,010; 0.276	

Clinical Frailty Scale

Parameter	Model 1 B (95% CI)	p	Model 2 B (95% CI)	p	Model 3 B (95% CI)	p
Age (years)	0.080 (0.070 – 0.089)	<0.001	0.079 (0.070 – 0.089)	<0.001	0.077 (0.066 – 0.087)	<0.001
Body mass index (kg/m²)	0.010 (0.000 – 0.021)	0.057	0.008 (−0.002 – 0.019)	0.120	0.009 (−0.002 – 0.020)	0.118
Serum albumin (g/dL)	−0.651 (−0.789 – −0.512)	<0.001	−0.629 (−0.783 – −0.475)	<0.001	−0.688 (−0.865 – −0.511)	<0.001
eGFR (mL/min/1.73 m²)	−0.003 (−0.005 – 0.000)	0.073	−0.002 (−0.005 – 0.000)	0.095	−0.002 (−0.005 – 0.001)	0.174
Haemoglobin (g/dL)	—	—	−0.019 (−0.056 – 0.019)	0.331	−0.004 (−0.044 – 0.036)	0.848
Male sex	—	—	−0.242 (−0.387 – −0.097)	0.001	−0.271 (−0.432 – −0.111)	<0.001
C-reactive protein (mg/dL)	—	—	—	—	0.021 (0.001 – 0.041)	0.040
VitD deficiency only	0.426 (0.282 – 0.569)	<0.001	0.434 (0.291 – 0.577)	<0.001	0.411 (0.254 – 0.567)	<0.001
B12 deficiency only	−0.060 (−0.331 – 0.211)	0.664	−0.036 (−0.306 – 0.235)	0.796	−0.094 (−0.407 – 0.218)	0.554

Parameter	Model 1 B (95% CI)	p	Model 2 B (95% CI)	p	Model 3 B (95% CI)	p
Co-deficiency	0.504 (0.242 – 0.767)	<0.001	0.533 (0.271 – 0.795)	<0.001	0.428 (0.125 – 0.731)	0.006
n; R^2	1,294; 0.354		1,294; 0.360		1,011; 0.383	

B – unstandardized regression coefficient; CI – confidence interval. Reference category: no deficiency. Model 1 adjusted for age, body mass index, serum albumin and eGFR; Model 2 additionally adjusted for haemoglobin and sex; Model 3 additionally adjusted for C-reactive protein. The reduced sample size of Model 3 reflects incomplete C-reactive protein data. Sex was entered as an indicator variable with male sex coded 1 and female sex coded 0; the coefficient therefore represents the difference for men relative to women. Em dash (—) indicates that the covariate was not included in that model. Abbreviations: eGFR – estimated glomerular filtration rate; MoCA – Montreal Cognitive Assessment; GDS – Geriatric Depression Scale; FRIED – Fried frailty phenotype.

Supplementary Table S2. Multiplicative interaction between vitamin D and vitamin B12 deficiency

Outcome	Term	B12 threshold < 200 pg/mL B (SE)	p	B12 threshold < 300 pg/mL B (SE)	p	n
MoCA	VitD deficiency	−1.549 (0.345)	<0.001	−1.356 (0.413)	0.001	1,246
	B12 deficiency	0.050 (0.653)	0.939	−0.219 (0.434)	0.615	
	VitD × B12 interaction	−0.850 (0.927)	0.360	−0.654 (0.635)	0.303	
GDS	VitD deficiency	0.266 (0.437)	0.543	−0.086 (0.523)	0.869	1,185
	B12 deficiency	0.285 (0.825)	0.730	−0.803 (0.546)	0.142	
	VitD × B12 interaction	1.505 (1.164)	0.196	1.506 (0.807)	0.062	
FRIED	VitD deficiency	0.347 (0.077)	<0.001	0.299 (0.092)	0.001	1,292
	B12 deficiency	−0.060 (0.146)	0.679	−0.128 (0.098)	0.190	
	VitD × B12 interaction	0.001 (0.204)	0.998	0.129 (0.142)	0.362	
Clinical Frailty Scale	VitD deficiency	0.426 (0.073)	<0.001	0.412 (0.088)	<0.001	1,294
	B12 deficiency	−0.060 (0.138)	0.664	−0.059 (0.092)	0.522	
	VitD × B12 interaction	0.139 (0.193)	0.472	0.085 (0.134)	0.526	

All models adjusted for age, body mass index, serum albumin and eGFR. In this specification the two deficiencies enter as separate binary terms together with their product, rather than as four mutually exclusive groups. A significant interaction term would indicate a supra-additive (synergistic) effect. The co-deficiency cell comprised 96 patients at the 200 pg/mL threshold and 278 patients at the 300 pg/mL threshold. Abbreviations: eGFR – estimated glomerular filtration rate; B – unstandardized regression coefficient; SE – standard error; MoCA – Montreal Cognitive Assessment; GDS – Geriatric Depression Scale; FRIED – Fried frailty phenotype.

Supplementary Table S3. Sensitivity analysis using the alternative vitamin B12 threshold of 300 pg/mL

Panel A. Kruskal–Wallis comparisons across the four groups

Instrument	Threshold < 200 pg/mL H	p	$ε^2$	Threshold < 300 pg/mL H	p	$ε^2$	n

Instrument	Threshold < 200 pg/mL H	p	ε²	Threshold < 300 pg/mL H	p	ε²	n
MoCA	59.18	<0.001	0.044	60.47	<0.001	0.045	1,279
GDS	10.51	0.015	0.006	12.25	0.007	0.008	1,213
FRIED	61.11	<0.001	0.043	65.96	<0.001	0.047	1,351
Clinical Frailty Scale	103.77	<0.001	0.075	104.84	<0.001	0.075	1,355
Edmonton Frail Scale	57.41	<0.001	0.043	59.22	<0.001	0.044	1,283
ADL	93.02	<0.001	0.067	98.13	<0.001	0.071	1,344
IADL	64.97	<0.001	0.089	65.32	<0.001	0.089	704

Panel B. Multivariable linear regression (Model 1 specification) using groups defined at the 300 pg/mL threshold

Outcome	Exposure group	B (95% CI)	p	n; R²
MoCA	VitD deficiency only	−1.356 (−2.166 – −0.546)	0.001	1,246; 0.289
	B12 deficiency only	−0.219 (−1.071 – 0.634)	0.615	
	Co-deficiency	−2.229 (−3.092 – −1.365)	<0.001	
GDS	VitD deficiency only	−0.086 (−1.113 – 0.940)	0.869	1,185; 0.049
	B12 deficiency only	−0.803 (−1.874 – 0.268)	0.142	
	Co-deficiency	0.617 (−0.485 – 1.719)	0.272	
FRIED	VitD deficiency only	0.299 (0.117 – 0.480)	0.001	1,292; 0.255
	B12 deficiency only	−0.128 (−0.319 – 0.064)	0.190	
	Co-deficiency	0.300 (0.107 – 0.493)	0.002	
Clinical Frailty Scale	VitD deficiency only	0.412 (0.241 – 0.584)	<0.001	1,294; 0.352
	B12 deficiency only	−0.059 (−0.241 – 0.122)	0.522	
	Co-deficiency	0.438 (0.255 – 0.621)	<0.001	

Group sizes at the 300 pg/mL threshold: no deficiency n = 471; isolated vitamin D deficiency n = 346; isolated B12 deficiency n = 264; co-deficiency n = 278. All regression models adjusted for age, body mass index, serum albumin and eGFR. Findings for cognition, frailty and functional status were confirmed at the alternative threshold, whereas the association with depressive symptomatology was attenuated and no longer significant. Abbreviations: H – Kruskal–Wallis test statistic, adjusted for ties; ε² – epsilon-squared effect size; B – unstandardized regression coefficient; CI – confidence interval; eGFR – estimated glomerular filtration rate; MoCA – Montreal Cognitive Assessment; GDS – Geriatric Depression Scale; FRIED – Fried frailty phenotype; ADL – Activities of Daily Living; IADL – Instrumental Activities of Daily Living.

Supplementary Table S4. Confirmation of the multivariable models using rank-transformed outcomes

Outcome	Exposure group	Model 1 (untransformed) B	p	Model 1 (rank-transformed) B	p	Concordant
MoCA	VitD deficiency only	−1.549	<0.001	−86.44	<0.001	Yes
	B12 deficiency only	0.050	0.939	6.67	0.861	Yes
	Co-deficiency	−2.348	<0.001	−137.95	<0.001	Yes
GDS	VitD deficiency only	0.266	0.543	11.90	0.600	Yes
	B12 deficiency only	0.285	0.730	8.87	0.836	Yes
	Co-deficiency	2.055	0.011	107.00	0.011	Yes
FRIED	VitD deficiency only	0.347	<0.001	96.03	<0.001	Yes
	B12 deficiency only	−0.060	0.679	−17.13	0.668	Yes
	Co-deficiency	0.287	0.043	75.41	0.052	Borderline
Clinical Frailty Scale	VitD deficiency only	0.426	<0.001	121.43	<0.001	Yes
	B12 deficiency only	−0.060	0.664	−4.79	0.894	Yes
	Co-deficiency	0.504	<0.001	139.49	<0.001	Yes

All models follow the Model 1 specification, adjusted for age, body mass index, serum albumin and eGFR. Rank transformation was applied to the dependent variable using mean ranks for tied values. Coefficients from rank-transformed models are expressed in rank units and are not interpretable in clinical units; only the direction and statistical significance are informative. Eleven of twelve coefficients retained their significance status; the coefficient for co-deficiency in the FRIED model moved marginally above the conventional threshold ($p = 0.043$ to $p = 0.052$). Abbreviations: B – unstandardized regression coefficient; eGFR – estimated glomerular filtration rate; MoCA – Montreal Cognitive Assessment; GDS – Geriatric Depression Scale; FRIED – Fried frailty phenotype.
